# Association of preoperative systemic inflammation with postoperative conduction block in TAVI patients

**DOI:** 10.3389/fcvm.2025.1671841

**Published:** 2025-10-03

**Authors:** Ying-Lu Shi, Dong-Sheng Di, Cheng-Xin Zhang, Wen-Hui Gong, Sheng-Lin Ge

**Affiliations:** ^1^ Cardiovascular Surgery Department, The First Affiliated Hospital of Anhui Medical University, Hefei, Anhui, China; ^2^ Department of Health Promotion and Behavioral Sciences, School of Public Health, Anhui Medical University, Hefei, Anhui, China

**Keywords:** conduction block, systemic inflammation, inflammatory biomarkers, neutrophil-to-lymphocyte ratio, risk stratification

## Abstract

**Background:**

Conduction block (CB) is a frequent complication following transcatheter aortic valve implantation (TAVI). Systemic inflammation may play a role in its development, but evidence is limited.

**Methods:**

This prospective study included 155 patients who underwent TAVI. Preoperative systemic inflammation markers—including systemic immune-inflammation index (SII), neutrophil-to-lymphocyte ratio (NLR), platelet-to-lymphocyte ratio (PLR), and lymphocyte-to-monocyte ratio (LMR)—were analyzed in relation to postoperative conduction block and cardiac function using logistic and linear regression models, as well as restricted cubic spline analysis.

**Results:**

Postoperative conduction block occurred in 35.5% of patients. Higher preoperative SII (OR = 1.0009; *P* = 0.0289), NLR (OR = 1.1630; *P* = 0.0253), and PLR (OR = 1.0079; *P* = 0.0065) were significantly associated with increased CB risk, while higher LMR was protective (OR = 0.7435; *P* = 0.0194). LMR was also independently associated with reduced ejection fraction and increased left ventricular volume. Gender subgroup analysis showed stronger associations in females.

**Conclusion:**

Preoperative systemic inflammation is independently associated with conduction block and cardiac function outcomes after TAVI. Inflammation-based biomarkers may serve as useful tools for risk stratification and perioperative planning.

## 1 Introduction

Severe aortic stenosis (AS) is a progressive and life-threatening valvular heart disease that predominantly affects elderly individuals (1). Transcatheter aortic valve implantation (TAVI) has become the treatment of choice for patients with symptomatic severe AS who are at high or prohibitive surgical risk (2, 3). Despite the minimally invasive nature and clinical success of TAVI, conduction disturbances remain a frequent and serious complication, with new-onset left bundle branch block (LBBB) (4), complete atrioventricular block (AVB) (5–7), and the subsequent need for permanent pacemaker implantation (PPMI) reported in a substantial proportion of patients (8, 9). These conduction abnormalities can negatively affect left ventricular remodeling, functional recovery, and long-term survival.

Emerging evidence suggests that systemic inflammation plays a critical role in cardiovascular remodeling, myocardial injury, and electrical conduction disturbances (10). Inflammatory biomarkers derived from routine blood tests—such as the systemic immune-inflammation index (SII), neutrophil-to-lymphocyte ratio (NLR), platelet-to-lymphocyte ratio (PLR), and lymphocyte-to-monocyte ratio (LMR)—have been shown to predict adverse outcomes in various cardiovascular settings, including coronary artery disease, heart failure, and postoperative complications in cardiac surgery (11–13). Among these markers, the NLR has been widely validated as a predictor of coronary artery disease (CAD) severity and long-term cardiovascular outcomes, including mortality and major adverse cardiovascular events (MACEs) (11, 13). The SII, which integrates neutrophil, platelet, and lymphocyte counts, has demonstrated superior prognostic performance in acute coronary syndrome and post-intervention settings (12, 14). Additionally, elevated PLR and decreased LMR have been associated with atherosclerotic burden and systemic inflammatory status, further supporting their value in cardiovascular risk stratification (15, 16). These markers provide insights into the balance between innate and adaptive immune responses and reflect the severity of systemic inflammation. However, data on the association between preoperative systemic inflammation and the risk of conduction block following TAVI are scarce and inconclusive.

Therefore, this prospective study aimed to evaluate the association between preoperative systemic inflammatory markers and the occurrence of postoperative conduction block in patients undergoing TAVI. We hypothesized that elevated levels of inflammatory indices such as SII, NLR, PLR, and decreased LMR are independently associated with an increased risk of conduction disturbances. Understanding this relationship may help identify high-risk patients preoperatively, optimize patient selection, and tailor perioperative monitoring strategies to improve outcomes after TAVI.

## 2 Method

### 2.1 Study population

This prospective, observational study included consecutive patients who underwent a TAVI for AS between September 2020 and April 2024 at the Department of Cardiac Surgery, The First Affiliated Hospital of Anhui Medical University. The inclusion criteria were: (1) patients with a confirmed diagnosis of severe AS who were deemed eligible for TAVI following a comprehensive evaluation by a multidisciplinary heart team; and (2) availability of preoperative laboratory data, including systemic inflammation markers such as SII, NLR, PLR, and LMR.

Patients were excluded if they (1) underwent surgical aortic valve replacement or other interventional treatments instead of TAVI, (2) had incomplete preoperative or postoperative data, or (3) experienced concomitant conditions that could significantly affect systemic inflammation levels (e.g., active infections, autoimmune diseases, or recent malignancies).

### 2.2 TAVI procedure

All TAVI procedures were performed at the Department of Cardiac Surgery, The First Affiliated Hospital of Anhui Medical University. Pre-TAVI evaluations included computed tomography, electrocardiography (ECG), and transthoracic echocardiography (TTE). All cases were discussed at a multidisciplinary team meeting to determine their suitability for TAVI.

The procedures were performed using standard bioprosthetic valve implantation techniques, and continuous ECG monitoring was performed for the first 24 h post-TAVI. Monitoring was extended for patients who exhibited significant conduction abnormalities. Routine postoperative evaluations included daily clinical assessments, TTE for cardiac function, and 12-lead ECGs during the hospital stay.

### 2.3 Systemic inflammation markers

Systemic inflammation markers, including the SII, NLR, PLR, and LMR, were calculated based on preoperative blood test results. Blood samples were collected from all patients within 24 h prior to the TAVI procedure as part of routine clinical evaluations.

The formulas for calculating these markers were as follows (17):

SII = (platelet count × neutrophil count)/lymphocyte count
NLR = neutrophil count/lymphocyte count
PLR = platelet count/lymphocyte count
LMR = lymphocyte count/monocyte count

All measurements were obtained from the hospital's clinical laboratory using standardized automated analyzers. The markers were analyzed as continuous variables in the primary analysis. These markers were chosen as they reflect different aspects of the systemic inflammatory response and have been associated with various clinical outcomes.

### 2.4 Postoperative cardiac outcomes and conduction block

The primary outcomes of this study were postoperative cardiac outcomes and postoperative conduction block.

Postoperative cardiac outcomes included ejection fraction (EF), left ventricular volume (LV), and left atrial size (LA), which were measured using transthoracic echocardiography (TTE) before TAVI and again prior to hospital discharge. Changes in EF, LV, and LA were calculated as the difference between the two measurements to evaluate postoperative cardiac function and structure.

Postoperative conduction block was defined according to standard ECG criteria, including:

1. new-onset complete atrioventricular block (third-degree AV block),
2. high-grade second-degree AV block (Mobitz type II with bradycardia),
3. new or worsening left bundle branch block (LBBB),
4. or other clinically significant conduction disturbances requiring permanent pacemaker implantation (PPMI).

All patients underwent continuous telemetry monitoring for at least the first 24 h post-TAVI, with extended monitoring in patients who developed conduction abnormalities. In addition, daily 12-lead ECGs were performed throughout the hospital stay to monitor the persistence or resolution of conduction block.

For patients requiring PPMI, implantation was performed during the same hospitalization, typically after ≥24 h of persistent conduction disturbance. The final decision was made by a multidisciplinary team consisting of the structural heart team and electrophysiology specialists.

### 2.5 Covariates

Potential confounding variables were carefully selected based on their clinical relevance and potential impact on the relationship between systemic inflammation markers and postoperative conduction block. The included covariates were age, gender, Euroscore2, smoking, drinking, residence (urban or rural), hypertension, diabetes, occupation group, and education group. Occupation group was categorized into three groups based on patients' self-reported employment status: non-agricultural workers, farmers, and retired individuals, to account for socioeconomic differences and lifestyle factors that may influence health outcomes. Education group was classified into three levels according to the highest level of education achieved: primary school or below, middle or high school, and college or above, as a proxy for socioeconomic status and health literacy. These covariates were incorporated into the multivariate logistic regression models to adjust for potential confounding effects and to isolate the independent association between systemic inflammation markers (e.g., SII, NLR, PLR, and LMR and postoperative outcomes.

### 2.6 Statistical analysis

All statistical analyses were performed using R software (version 4.3.1). Supplementary Table S1 shows missing data: height (14, 9.03%), weight (2, 1.29%), ICU stay time (1, 0.65%) had missing values; others (gender, age, hypertension, etc.) had none. Missing data were handled by imputing the mean for continuous variables and the mode for categorical variables. The normality of continuous variables was evaluated using the Shapiro–Wilk test. Parametric variables were presented as mean ± standard deviation (SD), while non-parametric variables were reported as median (interquartile range, IQR). Categorical variables were expressed as frequencies (percentages).

Patients were divided into two groups based on whether they developed conduction block post-TAVI. Data were compared between these cohorts using the following statistical tests: Student's *t*-test for parametric continuous variables, Mann–Whitney *U* test for non-parametric variables, *χ*² test, or Fisher's exact test for categorical variables as appropriate. The Wilcoxon signed-rank test was used for analyzing calcification distribution.

To explore the dose–response relationship between systemic inflammation markers and postoperative cardiac outcomes (including EF, LV, LA) and conduction block risk, we used restricted cubic spline (RCS) functions (18). The RCS method provides a flexible approach to model potential non-linear associations between continuous inflammation markers (such as SII, NLR, PLR, and LMR) and these outcomes. The analysis was performed using three knots placed at the 25th, 50th, and 75th percentiles of the continuous variable distributions. Visualizations were produced to assess the shape of the dose–response relationship for each inflammation marker in relation to both cardiac function and structure and conduction block risk.

Logistic regression was used to evaluate the association between preoperative systemic inflammation markers (e.g., SII, NLR, PLR, and LMR) and postoperative conduction block, with odds ratios (OR) and 95% confidence intervals (CI) reported. To assess the association between systemic inflammation markers and postoperative EF, LV, and LA, linear regression models were used. Regression coefficients (β) and 95% CI were reported.

A two-sided *P*-value < 0.05 was considered statistically significant. Data confidentiality prevents the public availability of study data.

## 3 Results

### 3.1 Baseline characteristics

Baseline characteristics of the study population stratified by the presence or absence of postoperative conduction block are presented in Table 1. A total of 155 patients undergoing TAVI were included, of whom 55 (35.5%) developed CB. There were no significant differences between the CB and non-CB groups in terms of demographic characteristics (age, BMI, residence, occupation, education), cardiovascular risk factors (hypertension, diabetes, smoking, drinking), or perioperative variables (Euroscore2, procedure time, ICU stay, total hospital duration) (all *P* > 0.05). In contrast, patients who developed CB exhibited significantly higher levels of SII (443.24 vs. 361.69; *P* = 0.048), NLR (2.71 vs. 2.18; *P* = 0.016), and PLR (135.26 vs. 110.88; *P* = 0.039), and lower LMR (3.38 vs. 3.83; *P* = 0.031), suggesting a potential association between heightened systemic inflammation and the development of conduction disturbances.

**Table 1:** Characteristics of participants.

Characteristics	Total (*N* = 155)	Conduction block		*P*-value
		No (*n* = 100)	Yes (*n* = 55)	
Age (years), mean ± SD	72.29 ± 6.73	72.60 ± 6.61	71.73 ± 6.96	0.449
Height (cm), mean ± SD	162.31 ± 7.95	161.86 ± 8.17	163.12 ± 7.56	0.337
Weight (kg), mean ± SD	61.03 ± 13.72	60.99 ± 11.03	61.10 ± 17.71	0.965
BMI (kg/m^2^), mean ± SD	23.19 ± 5.34	23.28 ± 3.77	23.04 ± 7.44	0.829
Smoking, *n* (%)	25 (16.13%)	18 (18.00%)	7 (12.73%)	0.531
Drinking, *n* (%)	19 (12.26%)	15 (15.00%)	4 (7.27%)	0.251
Residence, *n* (%)				0.196
Urban	27 (17.42%)	14 (14.00%)	13 (23.64%)	
Rural	128 (82.58%)	86 (86.00%)	42 (76.36%)	
Occupation, *n* (%)				0.663
Non-agricultural workers	36 (23.23%)	21 (21.00%)	15 (27.27%)	
Farmers	97 (62.58%)	64 (64.00%)	33 (60.00%)	
Retired	22 (14.19%)	15 (15.00%)	7 (12.73%)	
Education level, *n* (%)				0.318
Primary school or below	114 (73.55%)	77 (77.00%)	37 (67.27%)	
Middle or high school	37 (23.87%)	20 (20.00%)	17 (30.91%)	
College or above	4 (2.58%)	3 (3.00%)	1 (1.82%)	
Hypertension, *n* (%)	63 (40.65%)	43 (43.00%)	20 (36.36%)	0.526
Diabetes, *n* (%)	12 (7.74%)	7 (7.00%)	5 (9.09%)	0.755
Euroscore2	3.93 (1.92)	3.92 (1.60)	3.94 (2.41)	0.955
Surgery duration (mint)	146.46 (59.03)	145.79 (58.86)	147.67 (59.86)	0.851
ICU stay time (hours)	49.70 (95.34)	48.15 (86.61)	52.52 (110.26)	0.800
Total hospitalization duration (days)	23.56 (12.28)	22.39 (10.85)	25.69 (14.38)	0.141
SII	394.82 (276.54, 593.74)	361.69 (259.79, 556.54)	443.24 (306.17, 781.64)	0.048
NLR	2.40 (1.78, 3.45)	2.18 (1.72, 3.10)	2.71 (2.03, 4.60)	0.016
PLR	117.35 (88.81, 153.15)	110.88 (84.54, 147.13)	135.26 (95.10, 169.65)	0.039
LMR	3.58 (2.66, 4.80)	3.83 (2.76, 5.03)	3.38 (2.48, 4.09)	0.031

SD, standard deviation; BMI, body mass index; ICU, intensive care unit; SII, systemic immune-inflammation index; NLR, neutrophil-to-lymphocyte ratio; PLR, platelet-to-lymphocyte ratio; LMR, lymphocyte-to-monocyte ratio; Euroscore2, European system for cardiac operative risk evaluation II.

### 3.2 Dose–response relationship between system inflammation and conduction block risk

**Figure 1:**
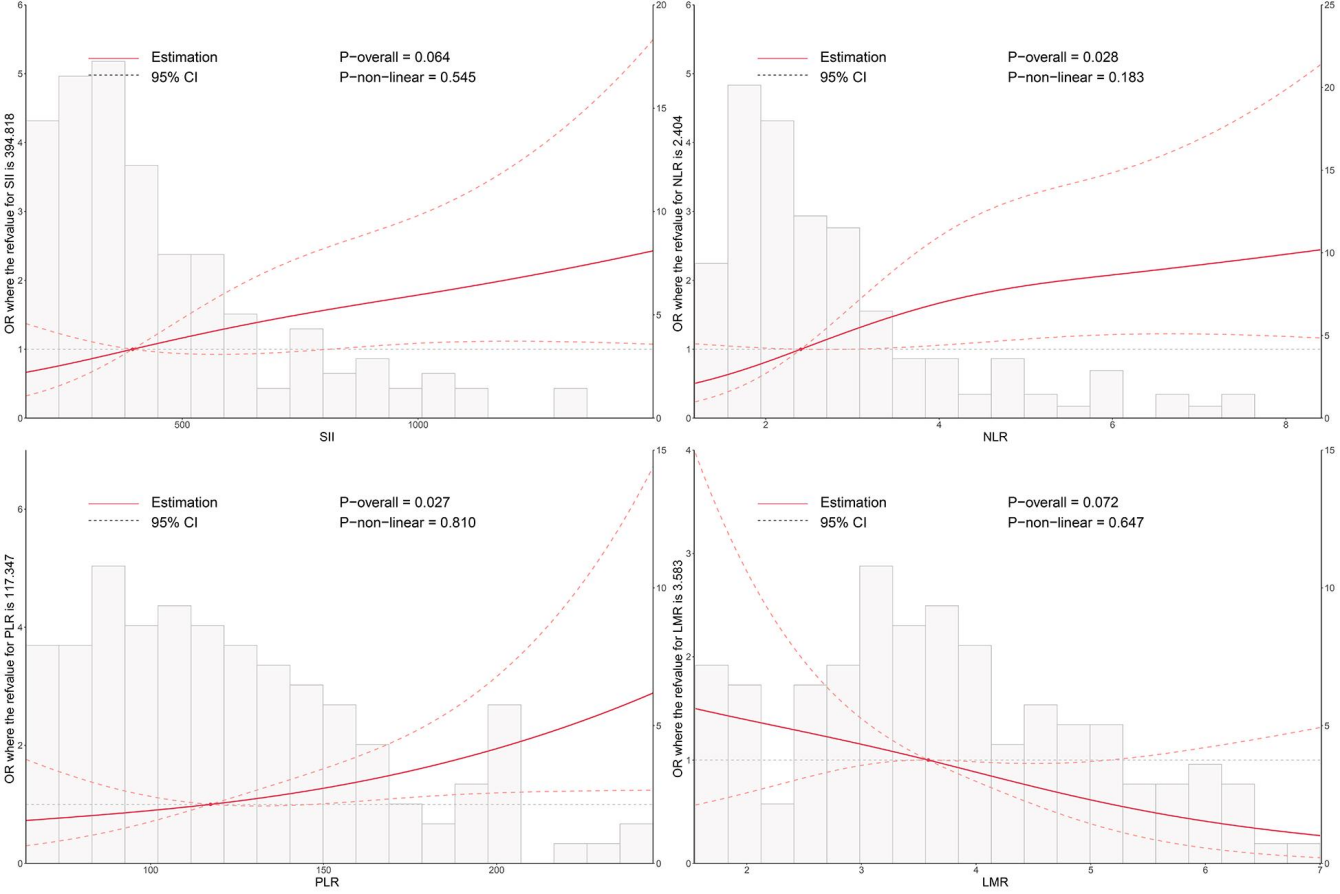
Dose–response relationship between systemic inflammatory markers and the risk of postoperative conduction block following TAVI. Restricted cubic spline (RCS) models were used to visualize the nonlinear associations of SII, NLR, PLR, and LMR with conduction block. The solid line represents the odds ratio (OR), and the shaded area indicates the 95% confidence interval (CI). SII, systemic immune-inflammation index; NLR, neutrophil-to-lymphocyte ratio; PLR, platelet-to-lymphocyte ratio; LMR, lymphocyte-to-monocyte ratio; TAVI, transcatheter aortic valve implantation.

As shown in Figure 1, RCS analysis revealed a positive association between higher levels of NLR and PLR and the risk of postoperative conduction block (*P*_overall_ = 0.028 and 0.027, respectively). SII showed a similar trend but did not reach statistical significance (*P*_overall_ = 0.064). LMR demonstrated a negative, though non-significant, association. These findings suggest a potential linear relationship between systemic inflammation and conduction disturbances after TAVI.

**Figure 2:**
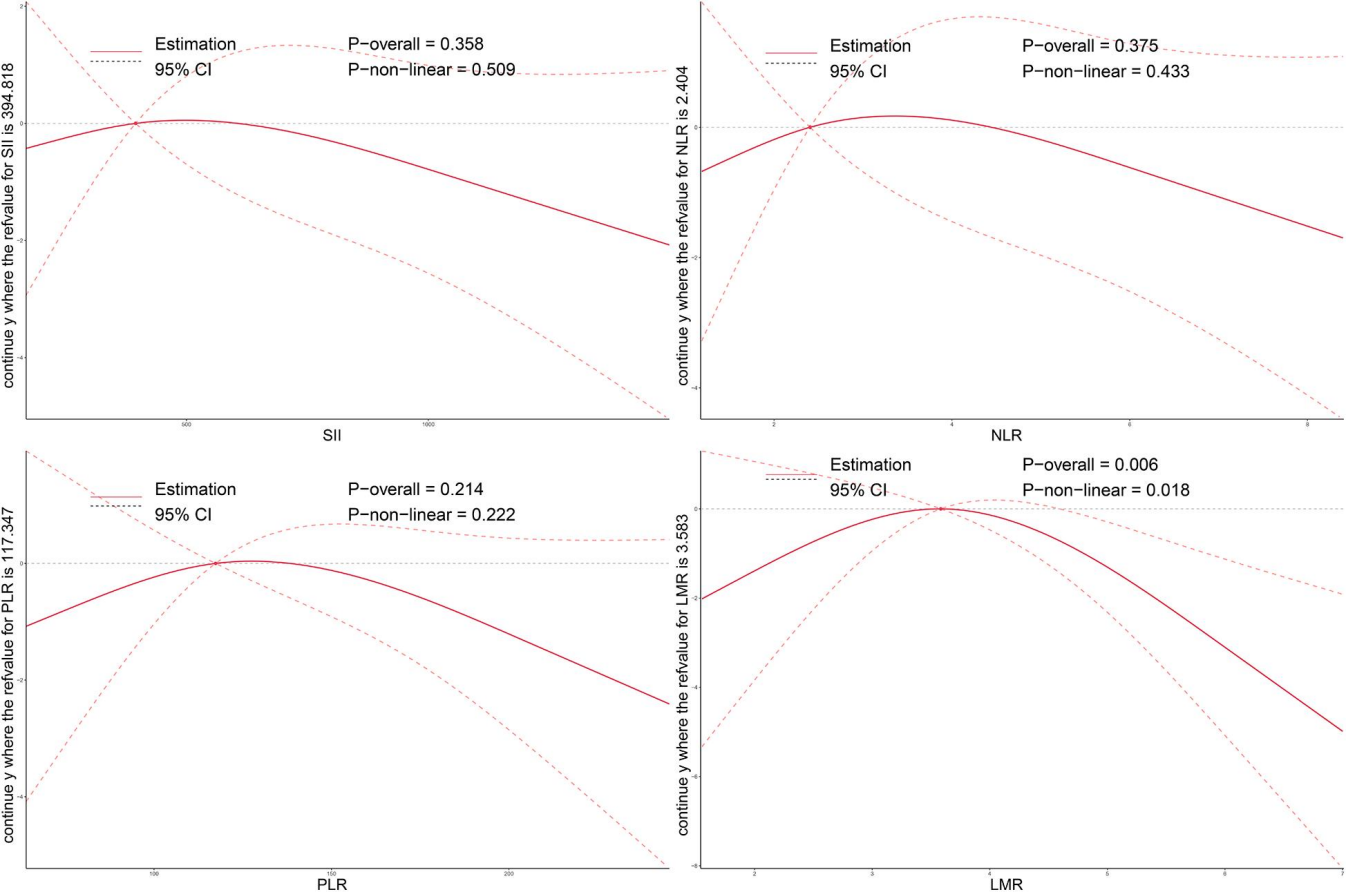
Dose–response relationship between preoperative systemic inflammatory markers and postoperative changes in EF after TAVI. Restricted cubic spline models were applied to explore the nonlinear associations of SII, NLR, PLR, and LMR with EF change. The solid line indicates the estimated effect, and the shaded area denotes the 95% confidence interval (CI). EF, ejection fraction; SII, systemic immune-inflammation index; NLR, neutrophil-to-lymphocyte ratio; PLR, platelet-to-lymphocyte ratio; LMR, lymphocyte-to-monocyte ratio; TAVI, transcatheter aortic valve implantation.

**Figure 3:**
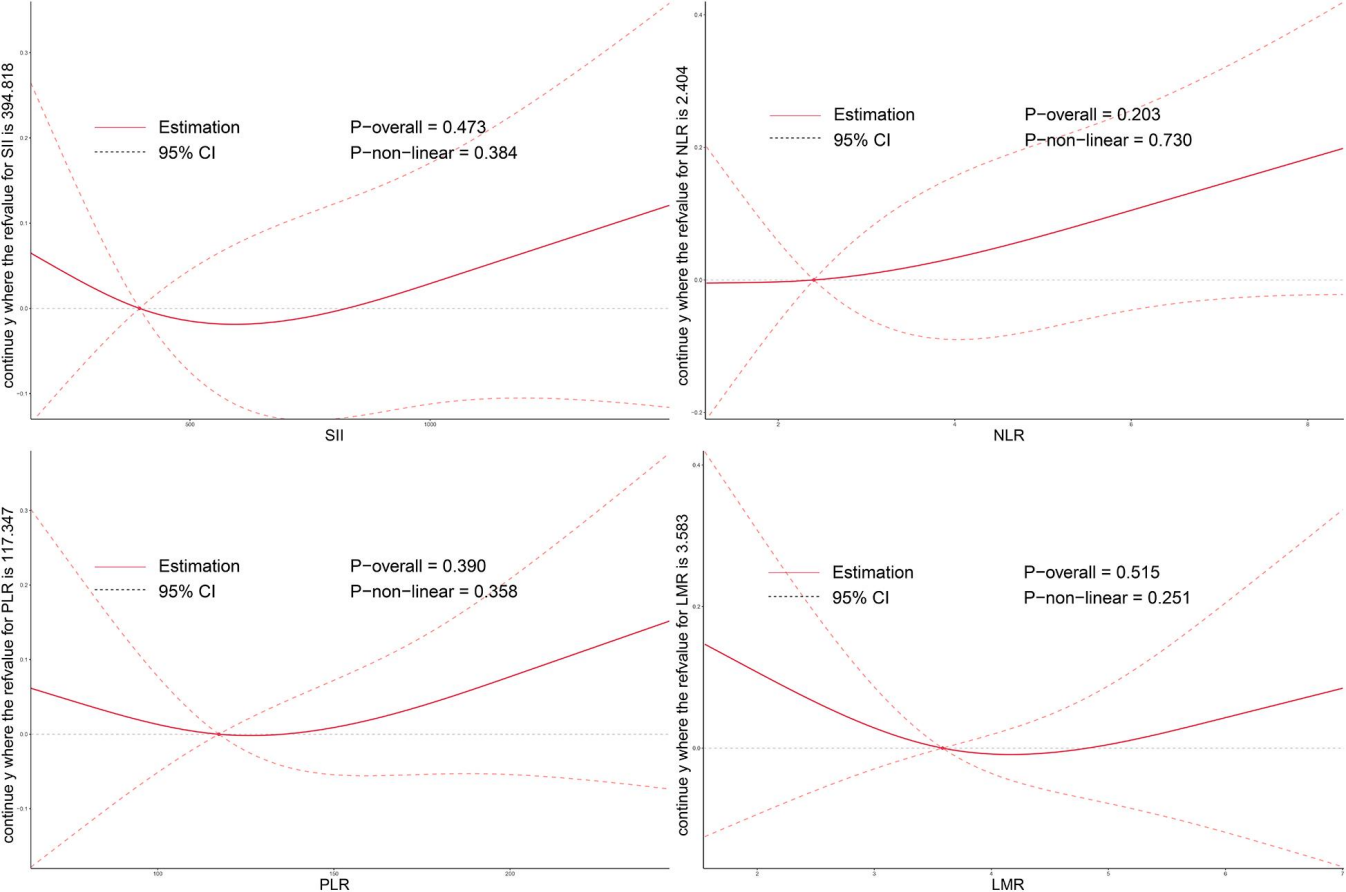
Dose–response relationship between systemic inflammatory markers and changes in LA after TAVI. Restricted cubic spline curves demonstrate the associations of preoperative SII, NLR, PLR, and LMR levels with postoperative LA change. LA, left atrial size; SII, systemic immune-inflammation index; NLR, neutrophil-to-lymphocyte ratio; PLR, platelet-to-lymphocyte ratio; LMR, lymphocyte-to-monocyte ratio; TAVI, transcatheter aortic valve implantation.

**Figure 4:**
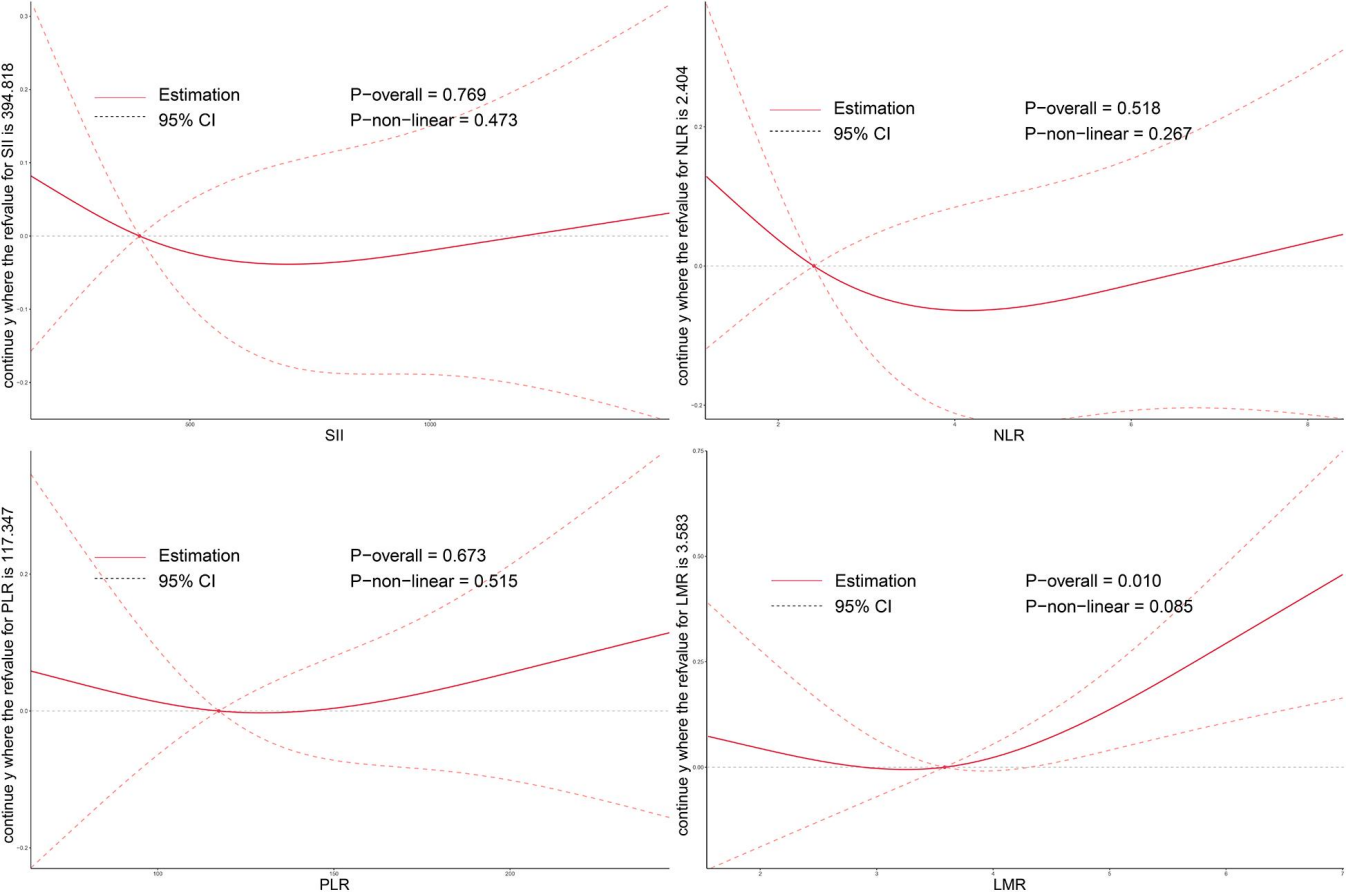
Dose–response relationship between systemic inflammatory markers and changes in LV after TAVI. RCS models were used to assess the associations between preoperative inflammation indices and postoperative LV remodeling. LV, left ventricular volume; SII, systemic immune-inflammation index; NLR, neutrophil-to-lymphocyte ratio; PLR, platelet-to-lymphocyte ratio; LMR, lymphocyte-to-monocyte ratio; TAVI, transcatheter aortic valve implantation.

As illustrated in Figures 2–4, no significant associations were observed between most inflammatory markers and postoperative cardiac function outcomes. An exception was LMR, which showed an inverse association with postoperative EF (*P*_overall_ = 0.006). No significant trends were found between any markers and changes in left atrial diameter or LV.

### 3.3 Associations between systemic inflammation and postoperative cardiac function and structure

Linear regression analysis results are summarized in Table 2. Among the systemic inflammation markers, only LMR showed statistically significant associations with multiple cardiac parameters. Higher preoperative LMR was significantly associated with lower postoperative EF (*β* = –0.7856; 95% CI: −1.4971 to −0.0741; *P* = 0.0321) and increased LV (*β* = 0.0870; 95% CI: 0.0199–0.1542; *P* = 0.0121). No significant associations were observed between LMR and LA. Other inflammatory markers—including SII, NLR, and PLR—showed no statistically significant associations with postoperative EF, LV volume, or LA size (all *P* > 0.05), although NLR exhibited a borderline trend with LA enlargement (*β* = 0.0327; *P* = 0.0790).

**Table 2:** Associations between systemic inflammation and postoperative cardiac function and structure.

System inflammation	EF	*P-*value	LV	*P-*value	LA	*P-*value
	*β* (95% CI)		β (95% CI)		β (95% CI)	
SII	−0.0017 (−0.0043, 0.0009)	0.2024	0.0000 (−0.0002, 0.0003)	0.9313	0.0001 (−0.0001, 0.0003)	0.3898
PLR	−0.0126 (−0.0320, 0.0069)	0.2077	0.0006 (−0.0013, 0.0024)	0.5450	0.0008 (−0.0007, 0.0024)	0.3078
NLR	−0.2730 (−0.7320, 0.1861)	0.2459	0.0062 (−0.0376, 0.0500)	0.7815	0.0327 (−0.0035, 0.0690)	0.0790
LMR	−0.7856 (−1.4971, −0.0741)	0.0321	0.0870 (0.0199, 0.1542)	0.0121	−0.0019 (−0.0594, 0.0556)	0.9485

SII, systemic immune-inflammation index; NLR, neutrophil-to-lymphocyte ratio; PLR, platelet-to-lymphocyte ratio; LMR, lymphocyte-to-monocyte ratio; OR, odds ratio; CI, confidence interval; EF, ejection fraction; LV, left ventricular volume; LA, left atrial size.

In gender-stratified analyses (Supplementary Table S2), the associations between preoperative systemic inflammation and postoperative cardiac function parameters were largely consistent across male and female subgroups. Notably, the inverse association between LMR and EF remained in both sexes but did not reach statistical significance (*P* = 0.1989 in females; *P* = 0.1226 in males). However, among male patients, LMR was significantly associated with increased LA (*β* = 0.1045; 95% CI: 0.0157–0.1934; *P* = 0.0237), a relationship not observed in females (*P* = 0.1858). No other significant associations were identified in either subgroup for SII, PLR, or NLR with EF, LV, or LA measurements (all *P* > 0.1).

These findings suggest that LMR may be a more sensitive indicator of postoperative structural changes, particularly in men, although the effect sizes were modest and warrant further validation in larger cohorts.

### 3.4 Associations between system inflammation and conduction block risk

Multivariable logistic regression analysis showed that all four systemic inflammatory markers were significantly associated with the risk of postoperative conduction block (Table 3). Higher levels of SII (OR = 1.0009; 95% CI: 1.0001–1.0016; *P* = 0.0289), PLR (OR = 1.0079; 95% CI: 1.0022–1.0137; *P* = 0.0065), and NLR (OR = 1.1630; 95% CI: 1.0189–1.3276; *P* = 0.0253) were independently associated with increased conduction block risk. Conversely, higher LMR was associated with reduced risk (OR = 0.7435; 95% CI: 0.5800–0.9532; *P* = 0.0194). These results suggest that elevated preoperative systemic inflammation may be a significant predictor of conduction disturbances following TAVI.

**Table 3:** Associations between system inflammation and conduction block risk.

System inflammation	OR (95% CI)	*P-*value
SII	1.0009 (1.0001, 1.0016)	0.0289
PLR	1.0079 (1.0022, 1.0137)	0.0065
NLR	1.1630 (1.0189, 1.3276)	0.0253
LMR	0.7435 (0.5800, 0.9532)	0.0194

SII, systemic immune-inflammation index; NLR, neutrophil-to-lymphocyte ratio; PLR, platelet-to-lymphocyte ratio; LMR, lymphocyte-to-monocyte ratio; OR, odds ratio; CI, confidence interval.

In the gender-stratified subgroup analysis (Supplementary Table S3), the associations were more pronounced in female patients. Among women, SII (OR = 1.0014; *P* = 0.0452), PLR (OR = 1.0114; *P* = 0.0127), and LMR (OR = 0.6345; *P* = 0.0451) remained significantly associated with conduction block risk, whereas NLR showed a borderline trend (*P* = 0.0842). In contrast, none of the associations reached statistical significance in the male subgroup (all *P* > 0.16). These findings indicate that systemic inflammatory markers may have stronger predictive value for conduction block in women than in men.

## 4 Discussion

In this prospective study of patients undergoing TAVI, we found that preoperative systemic inflammatory markers—including SII, NLR, and PLR—were positively associated with the risk of postoperative conduction block, while LMR was inversely associated. Notably, these associations appeared stronger among female patients. LMR was the only inflammatory marker significantly associated with postoperative changes in EF and LV. Additionally, among male patients, LMR was significantly associated with increased LA. These findings suggest that elevated systemic inflammatory markers, such as NLR and SII, could help identify patients at higher risk for postoperative conduction block. In clinical practice, this could guide extended ECG monitoring and closer follow-up for high-risk patients. Additionally, elevated inflammatory markers may prompt procedural adjustments, such as careful valve positioning, to minimize the risk of conduction disturbances during TAVI. These steps could improve personalized care and reduce procedural complications.

These results are consistent with prior studies showing the prognostic value of inflammation-based markers in cardiovascular diseases. In the context of TAVI, inflammation may contribute to conduction system vulnerability through several pathophysiological mechanisms. Neutrophils, as first responders to myocardial injury, are rapidly recruited to the site of infarction and release reactive oxygen species (ROS) and pro-inflammatory mediators, which aggravate tissue damage and promote fibrosis. They also form neutrophil extracellular traps (NETs) that contribute to local inflammation and cardiac injury (19). Although neutrophils also exert anti-inflammatory and reparative effects during later stages, their acute activation often amplifies myocardial injury (19). Platelets, meanwhile, are activated in pro-inflammatory conditions such as hypertension and atrial fibrillation, and release transforming growth factor-beta 1 (TGF-*β*1), which stimulates fibroblast proliferation and atrial fibrosis (20). Platelet–neutrophil interactions can further enhance inflammation through neutrophil necroptosis and NET release, promoting thrombosis and tissue damage (21). The interplay between neutrophils, platelets, and fibroblasts plays a central role in the development of cardiac fibrosis.

In addition, neutrophil-derived alarmins such as S100a8/a9 can activate cardiac fibroblasts, initiating inflammatory cascades that contribute to myocardial remodeling (22). The NLRP3 inflammasome within neutrophils further promotes myocardial injury via interleukin-1β (IL-1β) production and NET deposition (23). Beyond these cellular interactions, immune cells such as macrophages and lymphocytes are critical for clearing necrotic tissue and modulating the balance between pro- and anti-inflammatory responses during cardiac repair (24, 25). A skewed immune profile may exacerbate fibrotic remodeling and increase the risk of adverse outcomes.

Furthermore, systemic inflammation may disrupt autonomic regulation, contributing to electrophysiological instability. Evidence suggests that elevated inflammatory burden can suppress vagal tone and increase sympathetic activity, thereby reducing heart rate variability (HRV) and predisposing patients to adverse cardiovascular events. In endotoxemic models, sympathetic activation has also been shown to downregulate inflammatory responses, indicating a bidirectional interaction between inflammation and autonomic balance (26). Moreover, systemic inflammation is associated with carotid atherosclerosis, which can impair autonomic output and further exacerbate inflammatory dysregulation (27). These findings highlight the complex interplay between inflammation, autonomic dysfunction, and arrhythmogenic risk.

While the study found statistically significant associations between LMR and postoperative EF and LV volume, the effect sizes were relatively small (e.g., *β* = −0.7856 for EF). This suggests that LMR may not be a strong standalone predictor of cardiac outcomes in the postoperative setting. Instead, LMR may serve as a complementary marker that, when combined with other clinical and inflammatory variables, could provide additional insights into a patient's prognosis. However, given the modest effect sizes, the clinical utility of LMR in routine practice should be interpreted with caution. Further studies with larger cohorts and more robust validation are needed to determine whether LMR can be effectively integrated into risk stratification models for patients undergoing TAVI or other cardiovascular procedures.

This is one of the first studies to comprehensively evaluate the associations between multiple systemic inflammatory indices and both electrophysiological and structural outcomes after TAVI. Unlike previous studies that focused primarily on anatomical or procedural predictors, our study introduces routinely available inflammatory biomarkers—SII, NLR, PLR, and LMR—as novel predictors of conduction block. The use of restricted cubic spline modeling allowed for detailed assessment of potential non-linear relationships, enhancing the interpretability of the dose–response patterns. In addition, subgroup analyses by sex revealed important gender-specific differences that may inform personalized care. The stronger associations observed in females may be due to hormonal influences and higher baseline inflammation, which could contribute to more significant myocardial remodeling and conduction disturbances post-TAVI. These differences highlight the need for sex-specific strategies in TAVI. Future studies should explore how gender-related factors can refine risk assessment and personalize treatment to improve outcomes for female patients. Based on a real-world cohort with comprehensive perioperative data, our findings provide clinically actionable insights for refining preoperative risk stratification, tailoring ECG monitoring, and anticipating pacemaker needs, ultimately optimizing postoperative care and resource allocation.

Several limitations of this study should be acknowledged. First, this was a single-center prospective cohort study, which may limit the generalizability of our findings. Future multicenter studies with larger sample sizes are needed to confirm the external validity. Second, although we adjusted for multiple clinically relevant confounders, the possibility of residual or unmeasured confounding remains, especially regarding anatomical and procedural factors such as valve–septum distance, implantation depth, oversizing, and valve type and sizing, which were not systematically collected in this cohort. These variables are known predictors of conduction block, and their absence in this study may limit comprehensive risk stratification. The lack of these predictors also highlights the potential complementary value of inflammatory markers in predicting conduction block risk. Third, systemic inflammatory markers were measured only once within 24 h before the procedure, without perioperative or postoperative serial profiling. Repeated measurements may better capture the dynamic relationship between systemic inflammation and conduction system injury. Fourth, postoperative conduction disturbances were assessed using standard continuous telemetry and daily 12-lead ECGs; advanced electrophysiological mapping or high-resolution ECG techniques were not applied, which may have underestimated subclinical abnormalities. Finally, the present analysis focused on in-hospital outcomes; long-term follow-up data such as pacemaker dependency, cardiovascular events, and late conduction disturbances were not available. Therefore, further multicenter, prospective studies incorporating detailed anatomical imaging, procedural parameters, advanced electrophysiological assessment, serial inflammatory profiling, and long-term follow-up are warranted to validate and extend our findings.

## 5 Conclusion

In conclusion, this study demonstrates that elevated preoperative systemic inflammatory markers—including SII, NLR, and PLR—are significantly associated with an increased risk of conduction block following TAVI, while higher LMR appears protective. These associations are particularly pronounced in female patients. Additionally, LMR is independently related to changes in postoperative cardiac function. These findings underscore the potential utility of inflammation-based biomarkers in preoperative risk stratification and individualized management strategies for patients undergoing TAVI.

## Data Availability

The raw data supporting the conclusions of this article will be made available by the authors, without undue reservation.
