# Supplementary material for: Association of preoperative systemic inflammation with postoperative conduction block in TAVI patients
Source: Front Cardiovasc Med. 2025 Oct 3;12:1671841. doi: 10.3389/fcvm.2025.1671841 (PMC12533275; doi:10.3389/fcvm.2025.1671841)
Supplement: Supplementary file 1 [file Table1.docx]

Table S1. Missing Data Summary of Variables.

| **Variable** | **Missing Count** | **Missing Percent (%)** |
| --- | --- | --- |
| Height (cm) | 14 | 9.03 |
| Weight | 2 | 1.29 |
| ICU stay time (hours) | 1 | 0.65 |
| Gender | 0 | 0 |
| Age | 0 | 0 |
| Hypertension | 0 | 0 |
| Diabetes | 0 | 0 |
| Smoking | 0 | 0 |
| Drinking | 0 | 0 |
| Surgery duration (mint) | 0 | 0 |
| Total hospitalization duration (days) | 0 | 0 |
| Residence | 0 | 0 |
| Occupation | 0 | 0 |
| Education level | 0 | 0 |
| Preoperative WBC | 0 | 0 |
| Preoperative neutrophil count | 0 | 0 |
| Preoperative lymphocyte count | 0 | 0 |
| Preoperative monocyte count | 0 | 0 |
| Preoperative platelet count | 0 | 0 |
