# Supplementary material for: Association of preoperative systemic inflammation with postoperative conduction block in TAVI patients
Source: Front Cardiovasc Med. 2025 Oct 3;12:1671841. doi: 10.3389/fcvm.2025.1671841 (PMC12533275; doi:10.3389/fcvm.2025.1671841)
Supplement: Supplementary file 2 [file Table2.docx]

Table S2. Associations between systemic inflammation and postoperative cardiac function and structure by gender subgroup.

| Subgroup | System inflammation | EF | |  | LV | |  | LA | |
| --- | --- | --- | --- | --- | --- | --- | --- | --- | --- |
|  |  | β (95% CI) | *P-*value |  | β (95% CI) | *P-*value |  | β (95% CI) | *P-*value |
| Female | SII | -0.0021 (-0.0051, 0.0010) | 0.1867 |  | 0.0001 (-0.0002, 0.0004) | 0.4057 |  | 0.0001 (-0.0002, 0.0005) | 0.4405 |
|  | PLR | -0.0081 (-0.0305, 0.0143) | 0.4824 |  | 0.0004 (-0.0016, 0.0025) | 0.6756 |  | 0.0006 (-0.0018, 0.0030) | 0.6425 |
|  | NLR | -0.3326 (-0.8761, 0.2108) | 0.2361 |  | 0.0410 (-0.0075, 0.0894) | 0.1039 |  | 0.0292 (-0.0297, 0.0881) | 0.3354 |
|  | LMR | -0.6985 (-1.7496, 0.3525) | 0.1989 |  | -0.0099 (-0.1065, 0.0866) | 0.8414 |  | 0.0775 (-0.0357, 0.1908) | 0.1858 |
| Male | SII | -0.0017 (-0.0065, 0.0031) | 0.5006 |  | 0.0000 (-0.0004, 0.0003) | 0.7844 |  | -0.0002 (-0.0006, 0.0002) | 0.3461 |
|  | PLR | -0.0223 (-0.0582, 0.0135) | 0.2249 |  | 0.0007 (-0.0019, 0.0032) | 0.6114 |  | 0.0001 (-0.0031, 0.0033) | 0.9439 |
|  | NLR | -0.1757 (-1.0437, 0.6924) | 0.6927 |  | 0.0017 (-0.0603, 0.0636) | 0.9584 |  | -0.0389 (-0.1157, 0.0378) | 0.3232 |
|  | LMR | -0.8096 (-1.8264, 0.2073) | 0.1226 |  | 0.0192 (-0.0543, 0.0927) | 0.6098 |  | 0.1045 (0.0157, 0.1934) | 0.0237 |

Note: SII, Systemic Immune, Inflammation Index; NLR, Neutrophil, to, Lymphocyte Ratio; PLR, Platelet, to, Lymphocyte Ratio; LMR, Lymphocyte, to, Monocyte Ratio; OR, odds ratio; CI, confidence interval; EF, ejection fraction; LV, left ventricular volume; LA, left atrial size.
