# Supplementary material for: Association of preoperative systemic inflammation with postoperative conduction block in TAVI patients
Source: Front Cardiovasc Med. 2025 Oct 3;12:1671841. doi: 10.3389/fcvm.2025.1671841 (PMC12533275; doi:10.3389/fcvm.2025.1671841)
Supplement: Supplementary file 3 [file Table3.docx]

Table S3. Associations between system inflammation and conduction block risk by gender subgroups.

| subgroup | System inflammation | OR (95% CI) | *P-*value |
| --- | --- | --- | --- |
| Female | SII | 1.0014 (1.0000, 1.0027) | 0.0452 |
|  | PLR | 1.0114 (1.0024, 1.0204) | 0.0127 |
|  | NLR | 1.1715 (0.9789, 1.4020) | 0.0842 |
|  | LMR | 0.6345 (0.4066, 0.9900) | 0.0451 |
| male | SII | 1.0007 (0.9995, 1.0018) | 0.2772 |
|  | PLR | 1.0066 (0.9972, 1.0160) | 0.1678 |
|  | NLR | 1.1334 (0.9138, 1.4058) | 0.2544 |
|  | LMR | 0.8600 (0.6233, 1.1866) | 0.3585 |

Note: SII, Systemic Immune-Inflammation Index; NLR, Neutrophil-to-Lymphocyte Ratio; PLR, Platelet-to-Lymphocyte Ratio; LMR, Lymphocyte-to-Monocyte Ratio; OR, odds ratio; CI, confidence interval.
